# A novel nomogram based on the prognostic nutritional index for predicting postoperative outcomes in patients with stage I–III gastric cancer undergoing robotic radical gastrectomy

**DOI:** 10.3389/fsurg.2022.928659

**Published:** 2022-10-25

**Authors:** Danli Shen, Guowei Zhou, Jian Zhao, Gang Wang, Zhiwei Jiang, Jiang Liu, Haifeng Wang, Zhengming Deng, Chaoqun Ma, Jieshou Li

**Affiliations:** ^1^ Department of General Surgery, Jiangsu Province Hospital of Chinese Medicine, Affiliated Hospital of Nanjing University of Chinese Medicine, Nanjing, China; ^2^ Department of General Surgery, Jinling Hospital, Nanjing University, Nanjing, China

**Keywords:** prognostic nutritional index, prediction model, gastric cancer, enhanced recovery after surgery, robotic radical gastrectomy

## Abstract

**Background:**

The inflammation and nutrition status are crucial factors influencing the outcome of patients with gastric cancer. This study aims to investigate the prognostic value of the preoperative prognostic nutritional index (PNI) in patients with stage I–III gastric cancer undergoing robotic radical gastrectomy combined with Enhanced Recovery after Surgery (ERAS), and further to create a clinical prognosis prediction model.

**Study:**

525 patients with stage I–III gastric cancer who underwent ERAS combined with RRG from July 2010 to June 2018 were included in this work, and were divided randomly into training and validating groups in a 7-to-3 ratio. The association between PNI and overall survival (OS) was assessed by Kaplan-Meier analysis and the log-rank test. Independent risk factors impacting postoperative survival were analyzed with the Cox proportional hazards regression model. A nomogram for predicting OS was constructed based on multivariate analysis, and its predictive performance was evaluated using Harrell's concordance index (C-index), calibration plots, ROC curve, decision curve analysis (DCA), and time-dependent ROC curve analysis.

**Results:**

Survival analyses revealed the presence of a significant correlation between low preoperative PNI and shortened postoperative survival (*P *= 0.001). According to multivariate analysis, postoperative complications (*P *< 0.001), pTNM stage (II: *P *= 0.007; III: *P *< 0.001), PNI (*P *= 0.048) and lymph node ratio (LNR) (*P *= 0.003) were independent prognostic factors in patients undergoing ERAS combined with RRG. The nomogram constructed based on PNI, pTNM stage, complications, and LNR was superior to the pTNM stage model in terms of predictive performance. The C-indexes of the nomogram model were respectively 0.765 and 0.754 in the training and testing set, while AUC values for 1-year, 3-year, and 5-year OS were 0.68, 0.71, and 0.74 in the training set and 0.60, 0.67, and 0.72 in the validation set.

**Conclusion:**

Preoperative PNI is an independent prognostic factor for patients with stage I–III gastric cancer undergoing ERAS combined with robotic radical gastrectomy. Based on PNI, we constructed a nomogram for predicting postoperative outcomes of gastric cancer patients, which might be utilized clinically.

## Introduction

Gastric cancer (GC), as a malignancy originating from the epithelial cells of gastric mucosa, is the fifth most common cancer and the third leading cause of cancer mortality around the world (1). It is reported that there are over 1 million new cases of GC and nearly 800,000 GC-related deaths annually worldwide, of which 42.6% of new GC cases and 45% of GC-related deaths occur in China (1, 2).The onset of GC is insidious, once diagnosed, the majority of the patients are on the line of advanced stage (3). Although surgical resection is the only potentially curable modality of therapy for GC, the 5-year overall survival (OS) is below 50% (3). To this end, clinicians have taken a series of measures, including Enhanced Recovery after Surgery (ERAS), minimally invasive/robotic surgery, radiotherapy, and chemotherapy, to improve the long-term efficacy of radical gastrectomy (1).

An increasing number of experimental studies have shown that the postoperative survival and prognosis of GC patients depend not only on tumor-associated factors but also on host-specific factors, such as the preoperative nutritional status and the systemic inflammatory response (SIR). The prognostic nutritional index (PNI) reflects the preoperative nutritional and immune statuses of patients *via* the serum albumin level and lymphocyte counts. Since its initial report in 1984 by Onodera T et al. (4), numerous clinical studies have acknowledged its role as a major prognostic predictor of malignancies. The clinical researches on PNI in gastric cancer showed that, PNI was intimately related with disease-free survival and OS in patients with gastric cancer after radical gastrectomy (5). According to Xishan et al., PNI was positively correlated with pathological features of gastric cancer, such as lymphocyte infiltration, cancer differentiation and TNM stage, moreover, a low PNI might predict a poor prognosis in elderly patients with gastric cancer (6).

In the present study, we combined tumor-associated features and PNI to comprehensively evaluate the long-term survival benefits of patients who underwent robotic radical gastrectomy (RRG) combined with ERAS and predict the risk factors impacting patients' long-term survival. Based on these risk factors, a novel reliable prognostic scoring system was developed, with which the long-term outcomes of patients undergoing the aforementioned treatment regimen can be predicted effectively and accurately.

## Materials and methods

### Patients

**Figure 1:**
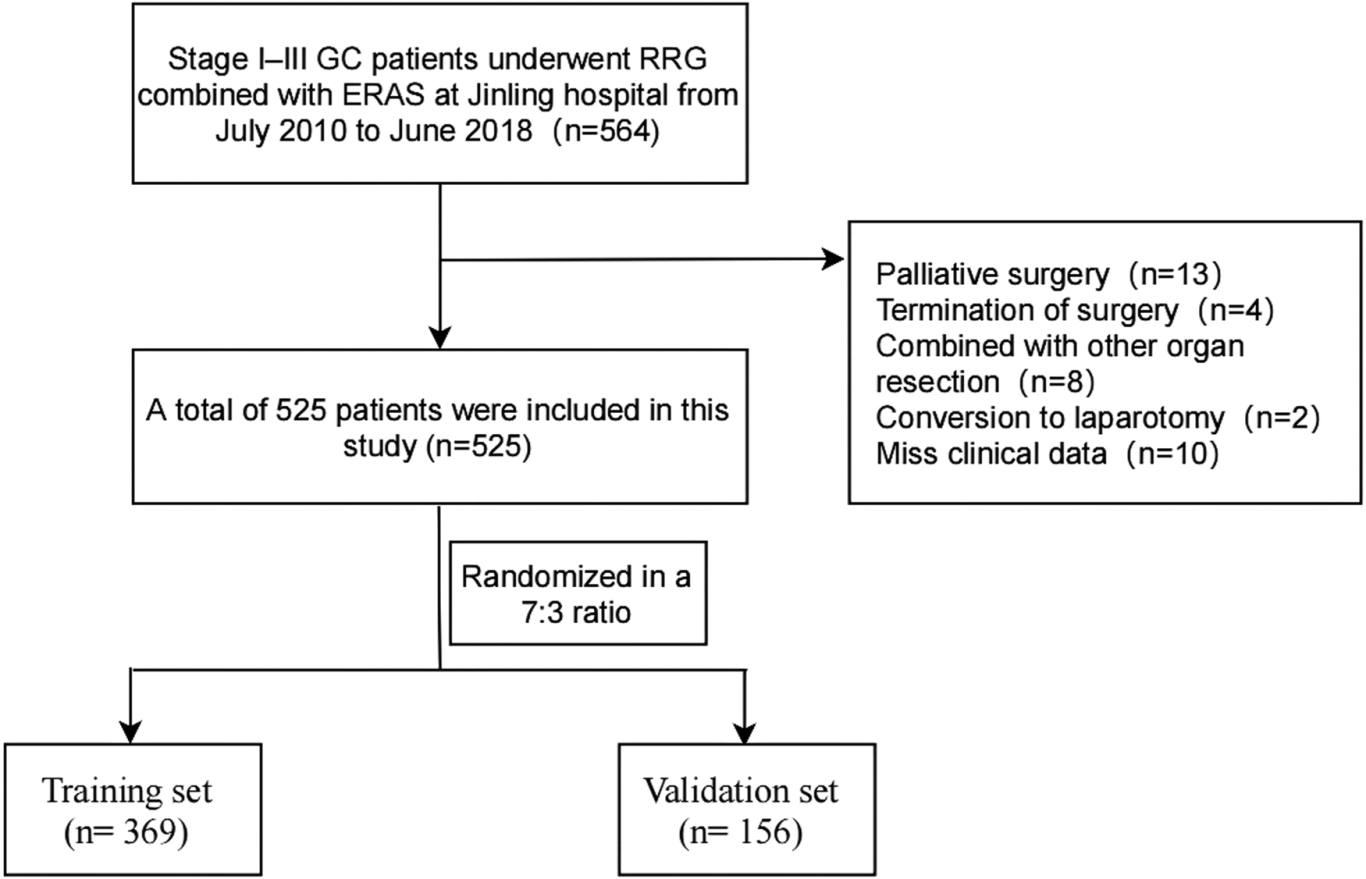
The flow diagram of stage I–III gastric cancer patients underwent robotic radical gastrectomy enrolled in this study. GC, gastric cancer; RRG, robotic radical gastrectomy.

We retrospectively analyzed the patients with stage I–III GC who underwent RRG combined with ERAS at the General Surgery Department of the Jinling Hospital from July 2010 to June 2018. The patients with stage II–III GC received neoadjuvant chemotherapy or postoperative chemotherapy, adopted the SOX regimen. The inclusion criteria were as follows: (1) diagnosed with gastric adenocarcinoma on preoperative gastroscopic histopathology; (2) absence of chronic hepatic/renal dysfunctions, infectious diseases, or immune system disorders; (3) no preoperative use of anti-infective agents; and (4) routine blood examination and C-reactive protein (CRP) and serum albumin measurements within 1 week preoperatively. The exclusion criteria were as follows: (1) gastric remnant cancer; (2) conversion to open laparotomy; (3) confirmed the diagnosis with extensive peritoneal seeding and/or distant organ metastasis; (4) palliative surgery; (5) gastrojejunal anastomosis; (6) combined resection of other organs intraoperatively; and (7) insufficient medical records. During this period, a total of 564 patients have received the treatment regimen. According to the inclusion and exclusion criteria, finally, 525 patients were enrolled in the present study and were randomized into the training set (*n* = 369) and the validation set (*n* = 156) at a ratio of 7:3 (see Figure 1). Relevant demographic information and clinical data were acquired by retrieving electronic medical records. The pathological tumor-node-metastasis (pTNM) classification was performed following the 8th edition of the American Joint Committee on Cancer (AJCC) staging system. The lymph node ratio (LNR) was defined as the ratio of the number of positive lymph nodes to the total number of retrieved lymph nodes. This study was approved by the Ethics Committee of Jinling Hospital and conformed to the principles of the Declaration of Helsinki.

### ERAS perioperative management measures

Under the guidance of Academician Jieshou Li of the Jinling Hospital combined with the years of clinical practice of Professor Zhiwei Jiang's team, a surgical clinical pathway to ERAS of GC, which consists of 17 regulations (details can be found in Supplementary Table S1), was established. All patients enrolled in this study followed the clinical management pathway.

### Laboratory indicators

Laboratory indicators, which were obtained within 1 week preoperatively, included neutrophil count, lymphocyte count, monocyte count, platelet count, CRP, and serum albumin. SIR indicators consisted of CRP, lymphocyte-to-monocyte ratio (LMR), neutrophil-to-lymphocyte ratio (NLR), and platelet-to-lymphocyte ratio (PLR). LMR was calculated by dividing the lymphocyte count by the monocyte count; NLR was calculated by dividing the neutrophil count by the lymphocyte count; PLR was calculated by dividing the platelet count by the lymphocyte count, and PNI was calculated by serum albumin (g/L) + 5 × lymphocyte count (×10^9^/L). The optimal cutoff values of LMR, NLR, PLR, and PNI were determined to be 3.05, 1.83, 207.3, and 45.39 (Supplementary Figures S1A–H) using X-tile 3.6.1 software (Yale University, New Haven, CT, USA) (7).

### Postoperative follow-up

All patients were subjected to routine follow-up at 1 month. They were followed up once every 3 months during the first 3 years postoperatively and once every 6 months during years 3–5 postoperatively, with the last follow-up on January 31, 2019. If a patient died or was lost to follow-up during this period, the follow-up was terminated. Follow-up consisted of physical examination, laboratory inspection (routine blood tests, hepatic/renal functions, tumor markers, etc.), and imaging examination (abdominal CT every 6 months, electronic gastroscopy every year). OS was defined as the period from the date of surgery to the date of the last follow-up (or to the date of termination of follow-up due to death or loss to follow-up).

## Statistical methods

R 4.1.0 software (https://www.r-project.org/) was used to perform data analysis. Continuous variables are expressed as the means ± SDs, while categorical variables are represented by numbers and percentages. Comparisons between continuous variables were made by unpaired *t*-tests, whereas comparisons between categorical variables were made by the chi-square test or Fisher's exact test. Kaplan-Meier analysis was employed to calculate OS, and the log-rank test was used to compare the survival curves. The independent risk factors impacting postoperative survival were identified through univariate and multivariate analyses with the Cox proportional hazards regression model. R software and the rms package were used to construct a nomogram, whose performance features were examined based on the generated calibration plot. The predictive performance of the nomogram model was evaluated using Harrell's concordance index (C-index), receiver operating characteristic (ROC) curves, decision curve analysis (DCA), and time-dependent ROC curve analysis. The prediction ability of the pTNM (Model A), no PNI-nomogram (Model B), and nomogram model (Model C) was evaluated by decision curve analysis. Two-tailed *P* values of <0.05 were regarded as significantly different.

## Results

### Baseline demographic characteristics

The median follow-up time was 41 months (range, 2–102 months) in the training set and 38 months (range, 1–101 months) in the validation set. The 3-year and 5-year OS rates were 80.9% and 74.8% in the training set, and 81.6% and 73.5% in the validation set. As shown in Table 1, in the analysis of the clinical characteristics of the patients included in the present study, there were no statistically significant differences between the training and validation sets (*P *> 0.05).

**Table 1:** Clinical characteristics of patients in the training set and validation set.

Characteristics	Training set (*n* = 369)	Validation set (*n* = 156)	*P*-value
Age, year	58.53 ± 10.14	57.87 ± 10.28	0.733
Sex			0.665
Male	274 (74.25%)	113 (72.44%)	
Female	95 (25.75%)	43 (27.56%)	
BMI, kg/m^2^	22.51 ± 2.89	22.81 ± 3.30	0.385
Neoadjuvant chemotherapy			0.297
No	292 (79.13%)	117 (75.00%)	
Yes	77 (20.87%)	39 (25.00%)	
Operation time, min	234.59 ± 33.30	237.28 ± 32.79	0.340
Blood loss, ml	92.90 ± 49.87	95.32 ± 53.30	0.762
Surgery			0.192
Proximal stomach	83 (22.49%)	27 (17.31%)	
Distal stomach	182 (49.32%)	90 (57.69%)	
Whole stomach	104 (28.18%)	39 (25.00%)	
Proximal margin, cm	4.82 ± 1.84	4.71 ± 1.91	0.482
Distal margin, cm	5.40 ± 1.62	5.43 ± 1.65	0.857
Postoperative hospital stay, d	6.60 ± 5.31	5.76 ± 3.39	0.053
Complications			0.943
No	328 (88.89%)	139 (89.10%)	
Yes	41 (11.11%)	17 (10.90%)	
Postoperative chemotherapy			0.798
No	180 (48.78%)	78 (50.00%)	
Yes	189 (51.22%)	78 (50.00%)	
Tumor size, cm	3.57 ± 2.16	3.41 ± 1.80	0.637
Tumor location			0.177
Upper	116 (31.44%)	42 (26.92%)	
Middle	146 (39.57%)	56 (35.90%)	
Lower	107 (29.00%)	58 (37.18%)	
Pathology			0.489
Well Differentiation	48 (13.01%)	26 (16.67%)	
Moderate Differentiation	160 (43.36%)	68 (43.59%)	
Poorly Differentiation	161 (43.63%)	62 (39.74%)	
Lymph node ratio	0.15 ± 0.24	0.13 ± 0.21	0.710
pTNM			0.450
I	138 (37.40%)	64 (41.03%)	
II	84 (22.76%)	39 (25.00%)	
III	147 (39.84%)	53 (33.97%)	
PNI			0.099
≤45.39	48 (13.01%)	29 (18.59%)	
>45.39	321 (86.99%)	127 (81.41%)	
LMR			0.446
≤3.05	106 (28.73%)	50 (32.05%)	
>3.05	263 (71.27%)	106 (67.95%)	
NLR			0.759
≤1.83	132 (35.77%)	58 (37.18%)	
>1.83	237 (64.23%)	98 (62.82%)	
PLR			0.674
≤207.30	331 (89.70%)	138 (88.46%)	
>207.30	38 (10.30%)	18 (11.54%)	
CRP, mg/L	3.13 ± 10.23	2.32 ± 4.59	0.733

pTNM, pathological tumor-node-metastasis; PNI, prognostic nutrition index; NLR, neutrophil-to-lymphocyte ratio; LMR, lymphocyte-to-monocyte ratio; PLR, platelet-to-lymphocyte ratio; CRP, C-reactive protein.

### Univariate and multivariate cox regression results

The univariate and multivariate Cox regression analyses, as demonstrated on Table 2, four parameters were independent prognostic factors: complications (Yes: hazard ratio (HR) 3.518, 95% confidence interval (CI) 1.974–6.269, *P *< 0.001), pTNM stage (II: HR 4.391, 95% CI 1.500–12.850, *P *= 0.007; III: HR 6.937, 95% CI 2.447–19.663, *P *< 0.001), PNI (>45.39: HR 0.553, 95% CI 0.306–0.993, *P *= 0.048) and LNR (4.349, 1.662–11.384, *P *= 0.003).

**Table 2:** Univariate and multivariate analysis of overall survival of patients undergoing RRG in the training set.

Characteristics	Univariate Analysis			Multivariate Analysis		
	HR	95% CI	*P*-value	HR	95% CI	*P*-value
Age, year	0.985	0.946–1.006	0.167			
Sex (male vs. female)	1.615	1.028–2.537	0.038*	1.511	0.941–2.428	0.088
BMI, kg/m^2^	0.980	0.907–1.060	0.619			
Neoadjuvant chemotherapy (No vs. Yes)	2.467	1.497–4.067	<0.001*	1.330	0.741–2.390	0.339
Operation time, min	0.997	0.991–1.004	0.375			
Blood loss, ml	1.001	0.997–1.005	0.633			
Surgery			<0.001*			0.056
Proximal stomach	Reference			Reference		
Distal tomach	1.950	1.400–2.717	0.556	1.803	0.885–3.673	0.105
Whole stomach	3.041	1.559–5.934	0.001*	2.458	1.176–5.137	0.017
Proximal margin, cm	1.040	0.925–1.168	0.514			
Distal margin, cm	0.918	0.809–1.042	0.186			
Postoperative hospital stay, d	1.020	0.989–1.052	0.218			
Complications (No vs. Yes)	2.536	1.486–4.327	0.001*	3.518	1.974–6.269	<0.001*
Postoperative chemotherapy (No vs. Yes)	2.294	1.434–3.669	0.001*	1.054	0.628–1.768	0.842
Tumor size, cm	1.191	1.094–1.297	<0.001*	1.028	0.925–1.144	0.606
Tumor location			0.677			
Upper	Reference					
Middle	1.200	0.696–2.068	0.512			
Lower	1.284	0.729–2.264	0.387			
Differentiation			<0.001*			0.169
Well	Reference			Reference		
Moderate	2.895	0.880–9.523	0.080	0.686	0.181–2.608	0.581
Poorly	6.053	1.890–19.388	0.002*	1.110	0.289–4.258	0.880
Lymph node ratio	10.715	5.499–20.878	<0.001*	4.349	1.662–11.384	0.003*
pTNM			<0.001*			0.001
I	Reference			Reference		
II	7.009	2.864–17.153	<0.001*	4.391	1.500–12.850	0.007*
III	9.355	4.020–21.768	<0.001*	6.937	2.447–19.663	<0.001*
PNI (≤45.39 vs. >45.39)	0.439	0.236–0.734	0.002*	0.553	0.306–0.993	0.048*
LMR (≤3.05 vs. >3.05)	0.564	0.362–0.877	0.011*	0.854	0.490–1.488	0.578
NLR (≤1.83 vs. >1.83)	1.948	1.177–3.222	0.009*	1.257	0.710–2.225	0.433
PLR (≤207.30 vs. >207.30)	1.708	0.944–3.090	0.077	0.775	0.399–1.504	0.451
CRP, mg/L	1.020	1.005–1.035	0.010*	1.001	0.984–1.019	0.882

pTNM, pathological tumor-node-metastasis; PNI, prognostic nutrition index; NLR, neutrophil-to-lymphocyte ratio; LMR, lymphocyte-to-monocyte ratio; PLR, platelet-to-lymphocyte ratio; CRP, C-reactive protein.

*P-value is less than 0.05, which is statistically significant.

### Construction and validation of the nomogram

**Figure 2:**
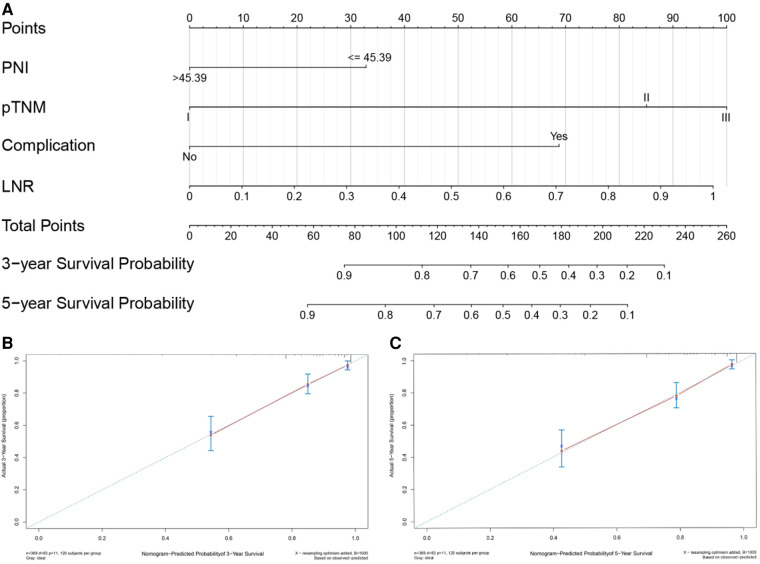
Nomogram for predicting 3- and 5-year overall survival (OS) of patients undergoing enhanced recovery after surgery (ERAS) combined with robotic radical gastrectomy. Nomogram for predicting the 3- and 5-year OS of patients undergoing ERAS combined with robotic radical gastrectomy (A). Calibration plot of the nomogram for (**B**) 3-year and (**C**) 5-year survival. PNI, prognostic nutrition index; LNR, Positive lymph node rate.

**Figure 3:**
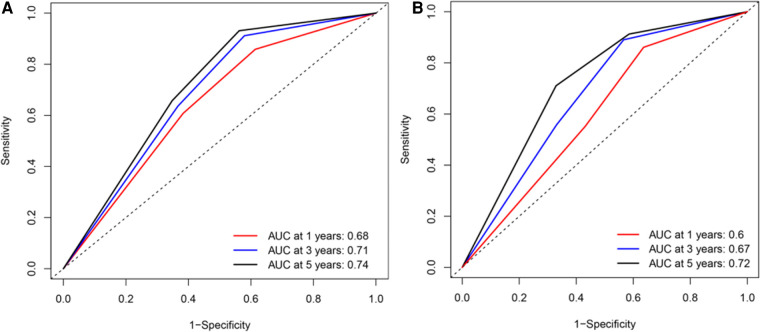
Receiver operating characteristic (ROC) curve for overall survival (OS) of patients undergoing enhanced recovery after surgery combined with robotic radical gastrectomy based on the nomogram. (**A**) ROC curve for 1-/3-/5- year OS based on the nomogram in the training set. (**B**) ROC curve for 1-/3-/5- year OS based on the nomogram in the validation set. AUC, area under the curve.

**Figure 4:**
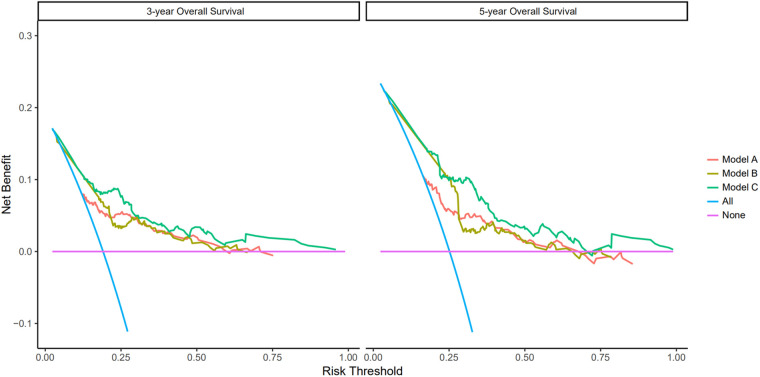
Prediction ability of the pTNM, no PNI-nomogram, and nomogram model for intensive overall survival (OS) of patients undergoing enhanced recovery after surgery (ERAS) combined with robotic radical gastrectomy in the training set. Decision curve analysis of 3- and 5-year OS of patients in the training set. The *X*-axis indicates the threshold probability for OS of patients undergoing enhanced recovery after surgery (ERAS) combined with robotic radical gastrectomy and the *Y*-axis indicates the net benefit. Compared to the pTNM, non PNI-nomogram, and nomogram model, the net benefit for the nomogram model was larger over the range of clinical threshold. [Model (**A**): the pTNM model; Model (**B**): the non PNI-nomogram model; Model (**C**): the nomogram model]. Abbreviations: AUC, area under the curve; PNI, prognostic nutrition index; pTNM, pathological tumor-node-metastasis.

Based on the results of univariate and multivariate Cox regression, four independent prognostic factors, namely, complications, pTNM stage, PNI, and LNR, were used to construct a nomogram (Figure 2A), and the C-indexes were 0.765 (95% CI 0.710–0.819) and 0.754 (95% CI 0.680–0.827) in the training and testing sets, respectively. Furthermore, the calibration plots (Figures 2B,C) indicated that the nomogram could precisely predict the 3-year and 5-year OS in the training set. The AUC values of the nomogram for 1-year, 3-year, and 5-year OS were 0.68, 0.71, and 0.74, respectively, in the training set and 0.60, 0.67, and 0.72 in the validation set (Figure 3A,B). Afterward, comparing the predictive value of the three prognostic models (Figure 4), the nomogram based on PNI was superior to the non-PNI nomogram and the pTNM stage model.

### Clinical utility of the nomogram

**Figure 5:**
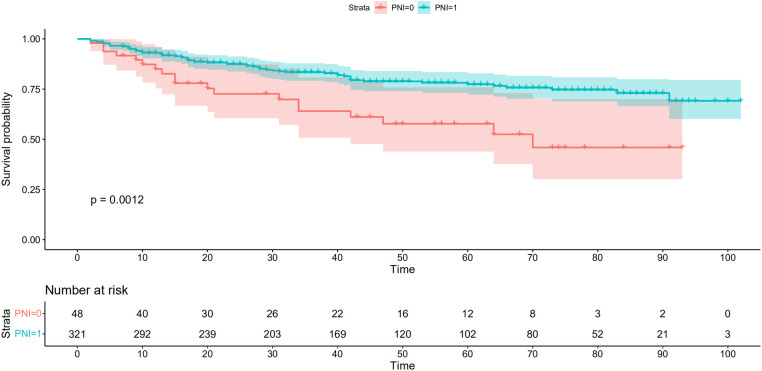
Kaplan-Meier analysis for overall survival (OS) of patients undergoing enhanced recovery after surgery combined with robotic radical gastrectomy according to the preoperative PNI. Red and lake blue solid lines represent Kaplan-Meier analysis for OS according to preoperative PNI ≤ 45.39 (PNI = 0) and PNI >45.39 (PNI = 1). PNI, prognostic nutrition index.

**Figure 6:**
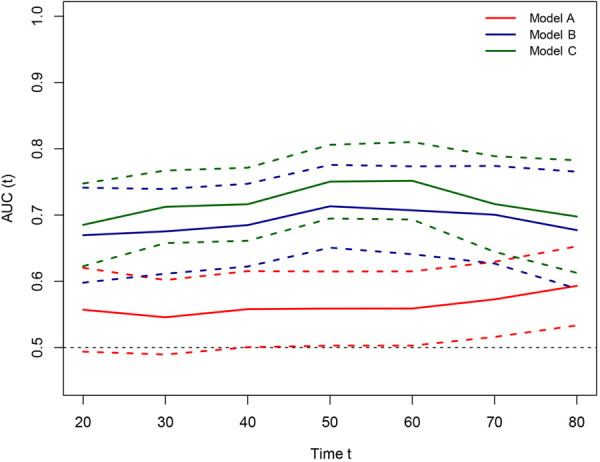
Time-dependent receiver-operating characteristic (ROC) curves for the pTNM, non PNI-nomogram, and nomogram model for the prediction of overall survival. The horizontal axis represents the year after surgery, and the vertical axis represents the estimated area under the ROC curve for survival at the time of interest. Red, blue, and green solid lines represent the estimated AUCs of the pTNM, no PNI-nomogram, and nomogram model; and broken lines represent the 95% confidence intervals of each AUC. [Model (**A**): the pTNM model; Model (**B**): the non PNI-nomogram model; Model (**C**): the nomogram model]. AUC, area under the curve; PNI, prognostic nutrition index; pTNM, pathological tumor-node-metastasis.

As shown in Figure 5, there were significant differences between the Kaplan–Meier survival curves of the two groups (*P *= 0.0012), which indicated that patients with PNI > 45.39 would have a significant survival advantage. Furthermore, the DCA curve (Figure 6) showed that compared with the non-PNI nomogram and the pTNM stage model, the nomogram based on PNI had a higher clinical application value and better clinical practicability in both the 3-year and 5-year analyses.

### Online model visualization

The online version of our nomogram is available at https://erasrobot.shinyapps.io/DynNomapp/. We hope to help more clinicians and research workers with this nomogram. After the user enters the clinical characteristics of the patient, he/she can easily predict the survival probability of the patient over time by reading the results and tables generated by the webserver.

## Discussion

Since the joint publication of the 1st edition of the *TNM Classification of Malignant Tumors* by the AJCC and the Union for International Cancer Control in 1968, the prognosis prediction and clinical treatment of GC patients have relied primarily on an evaluation system of TNM staging combined with histopathological classification. However, even if patients at the same pathological stages are treated with the same protocols, their prognostic outcomes can be quite different (8). This suggests that the current evaluation system is insufficient to accurately evaluate GC patient outcomes (9). Therefore, it is vital to further study the pathophysiological mechanisms of tumor progression, and identify individual risk factors (10).

To the best of our knowledge, the current study study firstly introduced a novel prognostic scoring system for the stage I–III GC patients who underwent RRG. The univariate and multivariate Cox analysis results indicated that PNI, pTNM stage, complications, and LNR were independent predictors for prognosis. A sensitive nomogram model was then constructed based on the above risk factors, and the prognostic model incorporating PNI (Model C) showed a higher predictive value than performance than TNM mode (Model A), and non PNI-nomogram (Model B).

An increasing amount of evidence has demonstrated that carcinogenesis and progression are extremely complex processes of pathological evolution that not only depend on the proliferation, invasion, and metastasis of cancer cells but are also regulated by the inflammatory immune response and nutritional status of hosts (11, 12). The signal of dystrophin can inhibit the activity of T cells *via* cytoplasmic nutrient sensors dependent signaling pathway, it also can destroy the intestinal villus and alter nutrient–sensing pathways, and thus decreases the immune function and the body's anti-tumor ability (13). A prospective cohort study among GC patients demonstrated that preoperative malnutrition and inflammatory increased the risk of postoperative complications (14). The postoperative nutritional management might improve the quality of life of people with radical gastrectomy. Although TNM stage is an important reference for the prognostic evaluation of cancer patients, an increasing number of clinical studies have recently demonstrated that novel prediction models based on PNI could further improve the staging of GC and achieve a better prediction of patient outcomes, which has profound clinical significance for guiding the individualized treatment of GC patients (15–18). As an easily obtainable biological indicator, PNI has been widely considered to dynamically reflect the immune function and nutritional status of the whole body (19). Since the innovative application of PNI by Onodera T for assessing the surgical risk and prognosis of patients with gastrointestinal malignancies, numerous studies (20–23), including our work, have demonstrated that PNI could be a significant prognostic factor for evaluating both short- and long-term outcomes of GC patients. Wang et al. (15) found that the fibrinogen and PNI (FPNI) score was significantly associated with advanced age, large tumor size, high American Society of Anesthesiologists (ASA) scores, late TNM stage, and poor prognosis (*P *< 0.01). According to multivariate analyses, preoperative PNI was an independent risk factor for disease progression and shortened survival in GC patients. A composite marker PNI-FPR, comprising PNI and fibrinogen-to-prealbumin ratio (FPR), when used in combination with the nomogram constructed based on T stage, pTNM, and surgery type, could preferably predict the postoperative prognosis of elderly GC patients (C-index = 0.742) (16). The inflammatory-nutritional prognostic score (INPS), composed of PNI and LMR, was an independent predictor of OS in stage III GC patients. The 5-year OS values of patients in the low, low-moderate, moderate-high and high INPS groups were 70.8%, 57.4%, 41.5% and 30.6%, respectively (17). Similarly, in patients with stage II–III GC who received postoperative chemotherapy, PNI was also found to significantly impact the disease outcome. In addition, compared to the pTNM model, the model constructed based on PNI, CRP-to-albumin ratio, percentage of preoperative weight loss, CA19–9, pTNM stage and tumor location could more accurately predict patient OS (C-index = 0.714 vs. 0.630, *P *< 0.001) (18). Similarly, this study also corroborated that lower preoperative PNI was associated with poorer postoperative prognosis for the stage I–III GC patients undergoing RRG combined with ERAS (HR 0.553, 95% CI 0.306–0.993, *P *= 0.048). Furthermore, the prognostic model incorporating PNI showed better performance than non PNI-nomogram, and TNM model, in predicting outcomes of GC patients undergoing RRG, of which the C-index was 0.765.

In addition, our results indicated that high LNR is also a significant prognostic parameter for poor patient prognosis (HR 4.349, 95% CI 1.662–11.384, *P *= 0.003). Lymph nodes are the transport hub of circulating immune cells and the portal for the malignant cell migration to and colonization of surrounding tissues and organs (24). Ikoma et al. reported that a high preoperative positive rate of central lymph nodes was significantly associated with late tumor stage and shortened OS, and a reduction in this positive rate by the preoperative anticancer treatment could improve the postoperative survival of patients (25, 26). According to a meta-analysis (27), the life expectancy of patients with a low LNR was significantly longer than that of patients with a high LNR (HR 1.99, 95% CI 1.72–2.27, *P *< 0.001). They verified this meta-model through sensitivity and regression analyses and called for the incorporation of the LNR into the future tumor staging system (27). Nakamura et al. (28) also reported that a high LNR was significantly correlated with a high risk of recurrence and metastasis following GC surgery and was an independent predictor of disease-free survival for GC patients at various stages. However, the above research failed to build prediction model incorporating LNR (29), while our present study filled this gap by establishing a novel stable LNR-incorporated model.

Among the 525 patients enrolled in the current study, 58 cases developed postoperative complications, including fever, infection, anastomosis, anastomotic stenosis, intestinal paralysis, delayed gastric emptying, intestinal obstruction, intestinal hernia, volvulus, pulmonary embolism, gastrointestinal bleeding, lymphatic leakage, duodenal stump fistula, and anastomotic fistula. The postoperative complication rate of patients was 11.0% in this study, which was lower than the reported 12.5–51.0% (30), reflecting the superiority of RRG combined with ERAS. Currently, it remains inconclusive whether postoperative complications can affect the outcome of GC patients. Chen et al.'s (30) meta-analysis revealed that the presence of postoperative complications severely shortened the life expectancy of patients with GC (HR 1.49, 95% CI 1.33–1.67, *P *< 0.001). In contrast, according to the results of Song et al. (31), postoperative complications in patients with stage II GC did not affect their tolerance to postoperative chemotherapy or their OS, and among patients with stage III GC, the presence of severe postoperative complications reduced their tolerance to chemotherapy and increased their risk of exacerbation. Our results showed that postoperative complications were an independent predictor of survival for GC patients (HR 3.518, 95% CI 1.974–6.269, *P *< 0.001). This may be attributed to the reduced incidence of minor complications by RRG combined with ERAS. The clinical studies demonstrated that RRG could reduce intraoperative blood loss, lower the incidence of major postoperative complications, and increase the number of lymph node dissections (32, 33).

There are some limitations in this study, which require further improvement. First, some clinical information and tumor heterogeneity features, such as smoking history, alcohol use, genomics biomarkers, the response to the radiochemotherapy and the history of H. pylori infection, were not included, which may lead to probable retrospective bias. Second, this study is a single center retrospective study, and multi-center and clinical prospective study is still required. Third, we failed to establish the correlation between our parameters and disease-free survival rate. Hence, the prognostic scoring system is needed to be further refined.

## Conclusion

In summary, our data demonstrated that, the easily obtainable and inexpensive biomarker PNI, was a significant prognostic factor for stage I–III GC patients undergoing RRG. Furthermore, incorporating preoperative PNI, pTNM stage, complications, and LNR, the further constructed nomogram had a better prediction accuracy and predictive performance than conventional models, which was conducive to guiding the individualized therapy of GC patients.

## Data Availability

The original contributions presented in the study are included in the article/**Supplementary Material**, further inquiries can be directed to the corresponding author/s.
