# Supplementary material for: A novel nomogram based on the prognostic nutritional index for predicting postoperative outcomes in patients with stage I–III gastric cancer undergoing robotic radical gastrectomy
Source: Front Surg. 2022 Oct 25;9:928659. doi: 10.3389/fsurg.2022.928659 (PMC9642802; doi:10.3389/fsurg.2022.928659)
Supplement: Supplementary file 1 [file Table1.pdf]

Table S1：The Clinical Pathway of Enhanced Recovery after Surgery for Radical Gastrectomy

| Period | Pathways |
|---|---|
| Preoperative | Health education: Attending doctors communicate the perioperative diagnosis and treatment plan to the patient and his family members, and conduct preoperative talks; full-time nurses explain to patients the methods of making or using drugs, guide functional exercise and diet.<br><br>Abstain from tobacco and drink: Quit smoking before surgery≥2 weeks; quit alcohol before surgery≥4 weeks.<br><br>Nutritional assessment: (1) Body mass index ＜18.5 kg/m$^2$; (2) Weight loss over the past 6 months＞10%; (3) Food intake decreased by 50% in recent one week; (4) Serum albumin＜30g/L. When any of the above items are met, the operation shall be postponed and preoperative nutritional support treatment shall be given first.<br><br>Preoperative fasting: Food deprivation 6 hours and water deprivation 2 hours before the operation. Drink 800ml of 12.5% carbohydrate drink at 20:00 the night before surgery, drink 400ml of 12.5% carbohydrate drink 2 hours before surgery.<br><br>Preoperative bowel preparation: Mechanical bowel preparation is not routinely performed before operation. Patients with chronic constipation are recommended to use a normal saline enema before surgery. |
| Intraoperative | Optimize anesthesia and sedation scheme: (1) Anesthesia induction: Midazolam 0.03mg/kg+ Sufentanil 0.03μg/kg+ Propofol 2mg/kg+ Rocuronium 0.6mg/kg face mask inhalatio, Medetomidine 0.6μg/kg intravenously pumped; (2) Anesthesia maintenance: Propofol 4~6mg/(kg·h) + Dexmedetomidine 0.2~0.4μg/(kg·h) + CIS-atracuramide 0.005~0.1mg/(kg·h) intravenously to continuously monitor the depth of anesthesia. If necessary, give sufentanil 10μg intravenously..<br><br>Strictly control the amount of intraoperative fluid infusion： Target-oriented fluid therapy is recommended to maintain the fluctuation of arterial blood pressure within ±20% of the basic value, or cardiac index > 2.5L / (min×m2).<br><br>Peform robot radical gastrectomy: (1) Adjust the surgical position: after general intravenous anesthesia, adjust the patient's position to a head-high-foot-low lying position (20°); (2)Establish pneumoperitoneum: place a 12 mm Trocar under the umbilicus to establish a pneumoperitoneum, maintain the pressure of the pneumoperitoneum at 10-12 mmHg; (3) Preoperative abdominal exploration: the robot lens enters through the Trocar hole to explore the abdominal cavity and rule out tumor seeding or distant metastasis; (4) Arrange the Trocar: insert an 8mm Trocar at the intersection of 2cm between the anterior axillary line on both sides and the lower edge of the rib, insert an 8mm Trocar at the intersection of the right clavicle midline and the horizontal line of the umbilicu, insert a 12mm Trocar at the intersection of the left mid-clavicular line and the horizontal line of the umbilicus; (5) |

| | |
|---|---|
| | Peform D2 lymph node dissection; (6) Gastrectomy: according to the location of the tumor, the stomach was cut off with a linear cutting and closure device and placed in the specimen bag, place the specimen bag temporarily in the pelvic cavity. (7) Digestive tract reconstruction: manual reconstruction of the digestive tract with 3-0 self-fixing knot-free thread under the microscope; (8) Take out the specimen: make a 4~6cm transverse incision on the symphysis pubis of the lower abdomen and take out the specimen bag.<br><br>Keep warm and cold-proof: during the operation, the warm blanket is covered, peritoneal lavage is performed with warm water at 37 ℃.<br><br>Unconventional placement of the nasogastric tube. |
